# Emotional Variance Analysis: A new sentiment analysis feature set for Artificial Intelligence and Machine Learning applications

**DOI:** 10.1371/journal.pone.0274299

**Published:** 2023-01-12

**Authors:** Leonard Tan, Ooi Kiang Tan, Chun Chau Sze, Wilson Wen Bin Goh

**Affiliations:** 1 School of Biological Sciences, Nanyang Technological University, Singapore, Singapore; 2 College of Engineering, Nanyang Technological University, Singapore, Singapore; 3 Lee Kong Chian School of Medicine, Nanyang Technological University, Singapore, Singapore; 4 Centre for Biomedical Informatics, Nanyang Technological University, Singapore, Singapore; Mae Fah Luang University, THAILAND

## Abstract

Sentiment Analysis (SA) is a category of data mining techniques that extract latent representations of affective states within textual corpuses. This has wide ranging applications from online reviews to capturing mental states. In this paper, we present a novel SA feature set; Emotional Variance Analysis (EVA), which captures patterns of emotional instability. Applying EVA on student journals garnered from an Experiential Learning (EL) course, we find that EVA is useful for profiling variations in sentiment polarity and intensity, which in turn can predict academic performance. As a feature set, EVA is compatible with a wide variety of Artificial Intelligence (AI) and Machine Learning (ML) applications. Although evaluated on education data, we foresee EVA to be useful in mental health profiling and consumer behaviour applications. EVA is available at https://qr.page/g/5jQ8DQmWQT4. Our results show that EVA was able to achieve an overall accuracy of 88.7% and outperform NLP (76.0%) and SentimentR (58.0%) features by 15.8% and 51.7% respectively when predicting student experiential learning grade scores through a Multi-Layer Perceptron (MLP) ML model.

## Introduction

Sentiment Analysis (SA) [1] is concerned with the measurement and classification of affective states in individuals; and is useful for inference related tasks like detection [2], recognition [3], recommendation [4], tracking [5], prediction [1], etc. SA has shown promise for use in science-of-learning (SOL) [6] and digital health [7] applications. For example, in SOL [6], SA has been applied in four major areas: (1) feature engineering, (2) learner engagement and satisfaction, (3) tutor teaching performance, (4) correlations between sentiment, behaviour, performance and achievement. In digital health (Healthcare 4.0) technologies [7], SA has been used to analyse social media data for (1) understanding sentiments of clinical practitioners towards policies, medication and rural health of the healthcare industry (2) studying patterns of communication about pain-related medical events, (3) detecting severity of medical symptoms (e.g. fibromyalgia) with weather variables.

SA can manifest as Text-Based Sentiment Tracking (TBST) [6], to detect affective states in text [5, 8]. TBST is the computational process of identifying whether a piece of writing expresses positive, negative or neutral attitudes and opinions in the context of a given topic in question [1]. This approach involves continuous batch-focused analyses around groups of structurally sequenced natural languages [1]. Examples include microblogs, recommendations, reviews, opinions, discussions, (email) correspondences, reports, documents, short messages, tweets, posts, etc. [4, 7].

Current TBST processes have evolved from two schools of thought: The first belong to the classical lexicon-based approaches [1, 9]. The second and more recent developments rely heavily on Machine Learning (ML) techniques [2, 5]. Lexicon-based approaches are sub divisible into dictionary and corpus-led categories [1], while ML-based techniques are sub divisible into supervised and unsupervised learning categories [10]. Furthermore, hybrid approaches–combining both lexicon and ML methods have been proposed in recent literature as efficient strategies [11].

The study of text-based feature extraction techniques in data analytics is an emerging trend [12, 13]. Current feature engineering approaches used in text classification face several key challenges [10, 14, 15]. Firstly, accuracy in sentiment classification techniques is not ubiquitous across structured, semi-structured and unstructured textual data [16–18]. Secondly, sparsity of textual data between words in a sentence lead to large error propagation at classifier boundaries [13]. Thirdly, sentiment polarity negation within a sentence of expressed sentiments may lead to feature bias during extraction [13]. Some current feature engineering techniques include: Imputation, Outlier handling, Binning (Discretization), Log Transform, One-Hot Encoding, Clustering, Feature Split, Scaling, Markovian Structuring, etc. [16–18]. Traditional Bag Of Words (BOW) and Part Of Speech (POS) tagging approaches, do not correlate semantics and concepts between words, thus highlighting the importance of novel feature engineering techniques [12, 13].

In feature engineering such as TBST, the goal of unsupervised feature selection is to automate the discovery of the smallest feature subsets which best uncovers informative clusters from textual data according to a discriminating objective [19–21]. However, unsupervised feature selection methods focuses on stochastic significance of features in maintaining word-lexicon data structures and ignores feature redundancy [1, 22, 23]. A diagram of feature selection methodologies is given in S3 Fig in S1 File. TBST suffers from two major drawbacks [6]. Firstly, sentiments which are identified from TBST processes at any of the abovementioned language specificity thresholds, tend to be scored and polarized at absolute values, which lacks information on sentiment dynamics [2]. Secondly, sentiment scores tend to be calculated from grouped or set averages, which lead towards gross over-averaging effects and concomitant loss of information [2, 5]. In this study, we address the above drawbacks by expanding calculations of sentiment polarity [11, 24, 25], orientation of the expressed sentiment (positive, negative or neutral) and sentiment polarity shifts–which can help capture significant emotional changes [26]. Our proposed feature set, Emotional Variance Analysis (EVA), captures and profiles changes in sentiment polarity and intensity to accurately classify emotional instability [10, 15, 20]. EVA comprises 21 novel EVA features calculated from extracted absolute rankings and sentiment scores based on observations of sentiment polarity shift profiles as relative polarity changes, through word-sentence vocabulary structures. EVA features in turn, are compatible as inputs for Artificial Intelligence (AI) and Machine Learning (ML) models. Indeed, studies have also shown that AI / ML models are sensitive to inputs, parameterization and tuning. Good initialization points at the input level are important contributors to AI/ML model performance. Thus, good feature engineering is important in establishing excellent initialization points through features with high information value for the learning process.

To illustrate EVA’s potential and applicability on real-world problems, we apply EVA on student journals captured over three years in an experiential learning course. We demonstrate that not only does EVA predict student academic performances accurately, it also outperforms existing natural language processing (NLP) features. We report that EVA is a powerful feature set and may see useful applications in other domains such as mental health profiling and consumer behaviour applications.

## Materials and methods

### Dataset

We evaluate EVA on student journals (also referred to as progress journals in the course) garnered from an experiential learning (EL) course. EL [6] is an educational construct that leverages on interactions with the environment to acquire knowledge through conceptualization, experimentation, experience and reflection [6]. To understand learner progress, a rich source of information are student journals, which captures both their emotional (motivation) states and learning gains (mastery) through the learning journey. This provides a window into students’ academic ability, learning gains and subject mastery [21]. Potentially, these journals could also be data mined for notions such as motivation [23], which are known to be positively correlated with good learning outcome [27].

The student journals are captured across a 13-week journey journey (which is also the full duration of an academic semester). On average, each student updates the journal once a week (sometimes more if they have been very active in their project work). The specific EL course under study here is known as the Nanyang Technological University (NTU) DEEP (Deeper Experiential Engagement Projects). DEEP runs between 2017–2019 are used for this study. While students were advised to write in a free-style manner, student journals can be broadly split into technical and reflective components. Technical components include project data and observations. Reflective components include personal thoughts, ideas and aspirations. Splitting journals into technical and reflective components also allowed us to test the hypothesis that EVA exerts its effectiveness primarily on reflective components.

Altogether, 37 individual DEEP students journals [28] constitute the dataset. Each are about 1.2MB in size with an average text length of 5000 words. The DEEP student journals were graded by 6 independent assessors at the end of the course, using rubrics which were designed for high Inter-Rater-Reliability (IRR) [29]. Assessor scoring was done based on assessment rubrics which were designed from theories of experiential learning [8]. Table 1 shows the grade distributions.

**Table 1:** Score distributions of student journals.

Deep Grade Distribution		
Grade	Score	Number Of Essays
**A**	> 81.59	6
**B**	76.43–81.59	9
**C**	71.27–76.43	17
**D**	<71.27	5

These journals are then assessed via SA and AI / ML techniques towards predicting student academic performance (see section on Multi-Layer Perceptron (MLP) Machine Learning Model). Model training and validation was based on 40-fold cross-validation: 80% (approximately 29 journals) were used in the training of our ML model while 20% (approximately 8 journals) were used in the testing of our ML model’s prediction performance. Earlier, we mentioned that there are 21 EVA features. These were applied on two variants of text: filtered and unfiltered for neutral sentences. In the former, filtering was conducted on the journals to remove sentences with a net polarity negation of zero for extraction of filtered EVA sentiment scores. In the latter, nothing was done. EVA features were extracted from both filtered and unfiltered texts for each journal, generating a feature vector of length 42 (21 EVA features for filtered and unfiltered versions of the same text respectively).

Given the grade distributions (Table 1), weighted K-nearest neighbour technique was used to determine the skew factor between population grade score mean and probability of occurrence. A Gaussian mask with these parameters was constructed with mean μ = 76.4 and standard deviation σ = 5.16.

### Other feature sets (for comparison)

Natural Language Processing (NLP) methods [30] facilitate analysis of text, providing some basic means for automated sentiment analyses. NLP techniques fall under two broad categories, statistical and neural approaches [31]. Statistical techniques require the use of distribution masks to make soft labelling decisions on sentiment classification and ranking [32]. Neural NLP-based approaches address the weakness of statistical NLP models by relying on error gradient inference mechanisms as a sequence-to-sequence transformation to extract, label and rank sentiment features [9].

The SpacY NLP package was used to extract a total of 56 features. These include tokenization (for segmenting text into words, punctuations, etc.), Part Of Speech / POS tagging (for assigning word types to tokens, like verbs or nouns), dependency parsing (which assigns syntactic dependency labels describing relations between lexicons), lemmatization (for semantic reduction of complex lexical representations into its base word form), named entity recognition / NER (which assigns labels to recognizable lexicons with real-world objects), entity linking / ER (for disambiguation of textual entities into unique identifiers for embedding into a knowledge base), etc.

SentimentR is a sentiment analysis package written in the "R" language [33]. It calculates sentiment polarity scores at individual sentence levels—including aggregated row and grouping variables [33]. It uses a dictionary-based approach by iteratively referencing a look up table (LUT) while also incorporating weighting for valence shifters [33]. Data handling and manipulation in SentimentR is supported by standard functions provided internally. Some key functions include nearest neighbour word clustering, negation (semantic inversion), amplification, de-amplification, adversative conjunctions, n-gram thresholding, word-2-vec, cosine-similarity, etc. SentimentR also enables extraction of sentiments at both sentence and group (of sentences) levels. SentimentR averages expresses sentiment scores of word-lexicon to sentence structures and sentence structures to group structures depending on the level of analysis involved.

Essentially, sentiment scores in SentimentR are are expressed as:

$$\delta =\frac{C^{\prime }}{\sqrt{\omega }}$$ (1)

**Fig 1:**
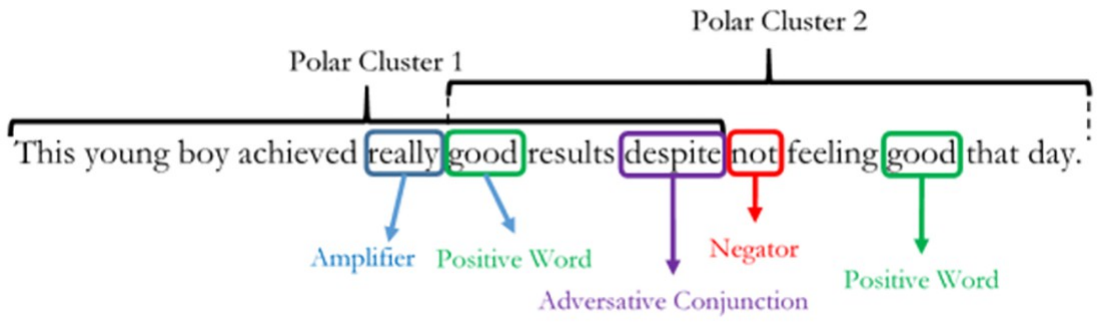
Sentence structure. The diagram shows a typical polarized sentence structure.

Where *δ* is defined as the sentiment score of the sentence. *C*′ is the sum of all weighted context clusters in the sentence and *ω* is the sentence length (word count). Context clusters are formed from a sequenced combination of valence shifters and polarized words. An example structure is given in Fig 1 below.

Nearest neighbour word clusters built around polarized key words define a polar cluster. In Fig 1, there are two polar clusters which co-exist within a single sentence structure. By default, each polar cluster comprises one polarized word (*W*^*P*^) with five words before and two words after the *W*^*P*^. Within a polar cluster, the types of valence shifters that SentimentR identifies are negators (*W*^*neg*^), amplifiers (*W*^*amp*^), de-amplifiers (*W*^*deamp*^) and adversative conjunctions (*W*^*ac*^). Negators are given by the following mathematical expression as:

$$W_{neg}=\left(\sum_{\;}^{\;}W^{neg}\right)mod2$$ (2)

**Fig 2:**
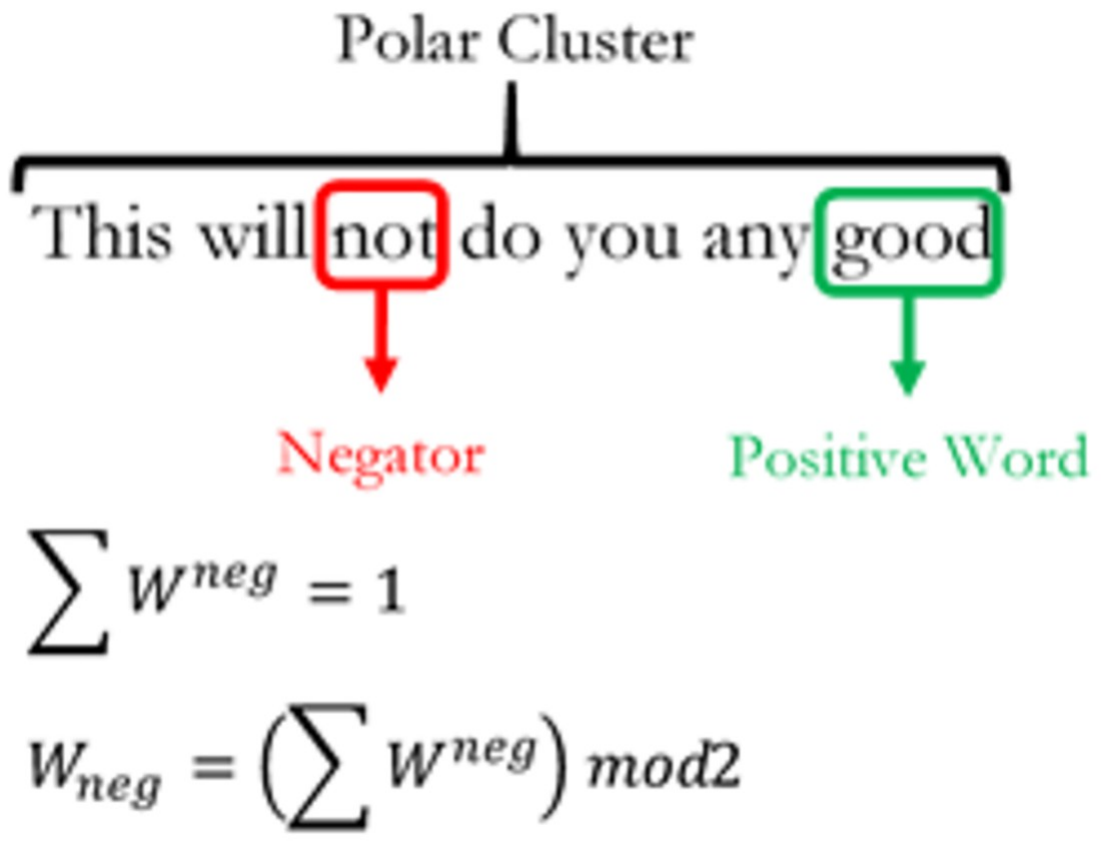
The net negation effect on the polarized word in a sentence cluster.

Eq (2) calculates the number of negators within a polar cluster taken to the modulo of 2. The result *W*_*neg*_ is either 1 or 0. A value of 1 implies an odd number of negators within the cluster and thus, there is a net negation effect on the polarized word. We provide an example on how negation functions work in Fig 2 below.

**Fig 3:**
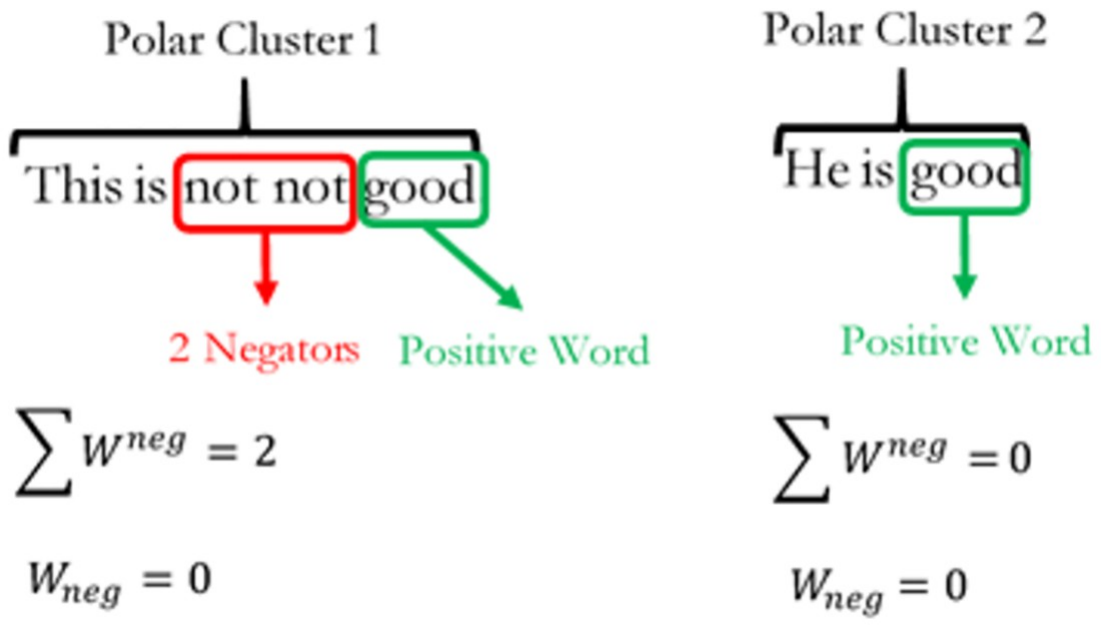
Negator cancellation effect. The diagram shows the net negation effects of words in a sentence.

When there are an odd number of negators; *W*_*neg*_ = 1, there is a net negation effect on the polar cluster in Fig 2. This means that the positively polarized word “good” in the sentence has been negated by the negator “not” to give a net sentiment polarity score of zero at the sentence level. However, if there are even factors of negators within a single polar cluster, then the net negation effect cancels out. An example of the negator cancellation effect is shown in Fig 3 below such that, a negating effect of 0 implies an even number of negators or the absence of negators within the cluster and thus contains no negation effect on the polarized word. Amplifiers and de-amplifiers are defined as adjacent word lexicons that either augments or attenuates sentiment polarity intensity respectively. Expressions of amplifiers and de-amplifiers are given as:

$$W_{deamp^{\prime }}=\sum_{\;}^{\;}z\left[\left(-W_{neg}.W^{amp}\right).W^{deamp}\right]$$ (3)

And

$$W_{amp^{\prime }}=\sum_{\;}^{\;}\left[\left(1-W_{neg}\right).z.W^{amp}\right]$$ (4)

Where *W*_*deamp*_ is the polarized word deamplifier, *W*_*amp*_ is the polarized word amplifier and *W*_*neg*_ is the polarized word negator.

**Fig 4:**
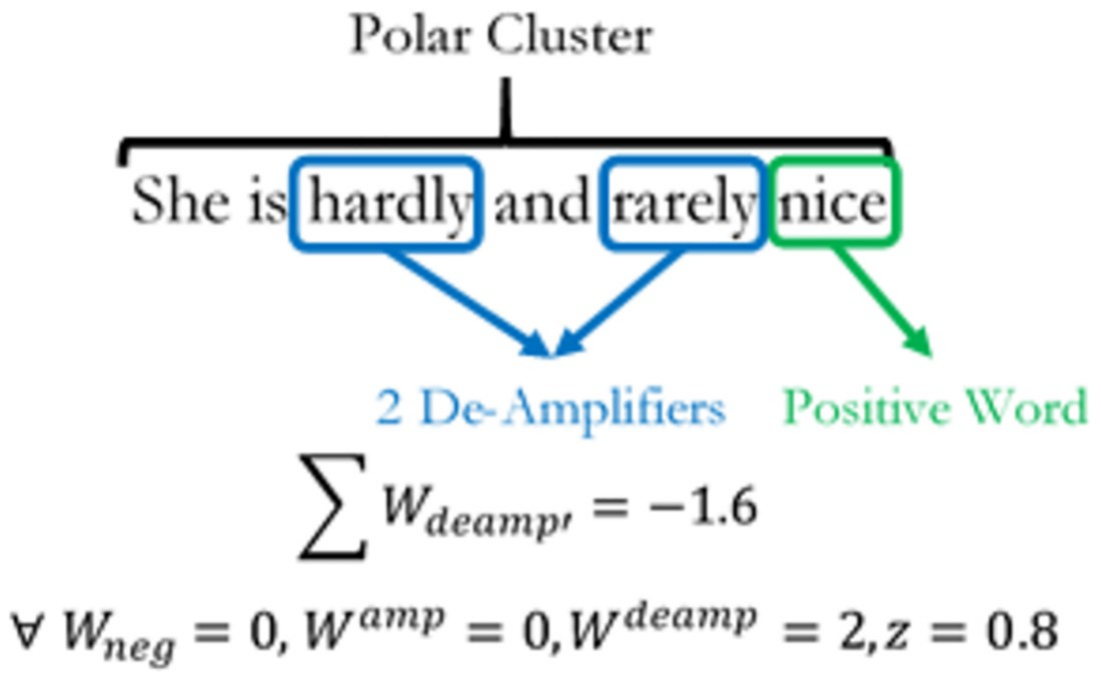
Positive de-amplification. The diagram shows the positive de-amplification of words in a sentence.

**Fig 5:**
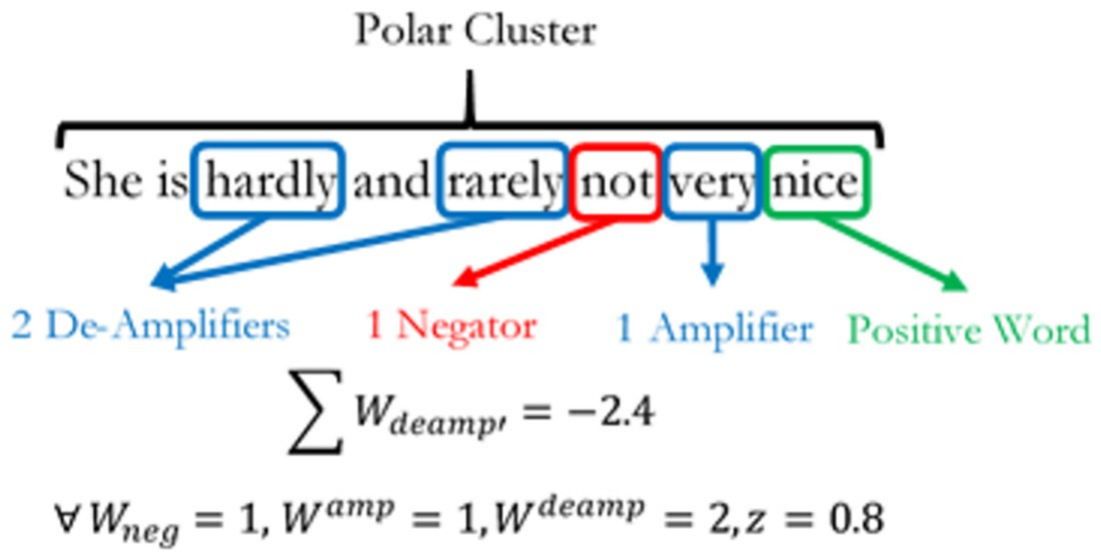
Negator de-amplification. The diagram shows the net negative amplification from a positive negation of words in a sentence.

**Fig 6:**
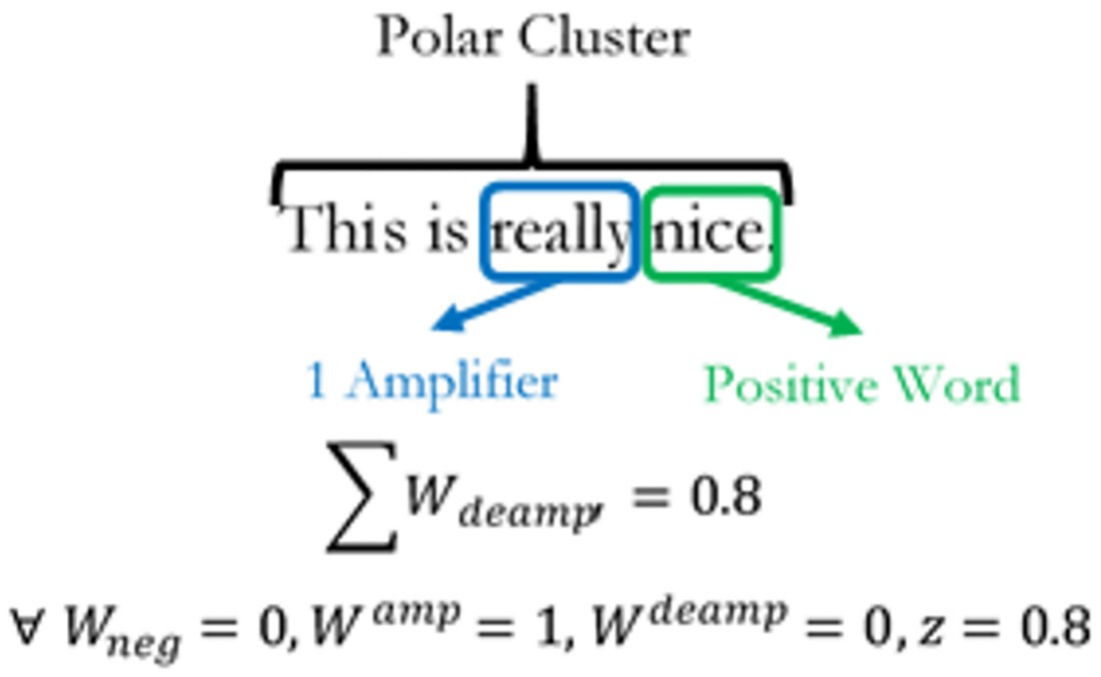
Positive amplification. The diagram shows the net positive amplification of words in a sentence.

Both equations consider net effects of *W*_*neg*_ on amplification and de-amplification within a cluster. Thus if *W*_*neg*_ is 0, then there is no negation effect on the amplifier. The number of de-amplifiers in the cluster will be multiplied by -1, weighted by a factor of *Z*, with a default value of 0.8 and summed together to give the value $W_{deamp^{\prime }}$. E xamples of how amplifiers and de-amplifiers function in SentimentR is given in Figs 4–6 below. Adversative Conjunctions (AC) are measures of contrasting oppositions within the sentence. They are defined as sentiments expressed in word clusters within a sentence that are polar opposites of each other. Mathematically, they are expressed as:

$$W_{ac}=1+Z_{ac}.\sum_{\;}^{\;}W^{ac}$$ (5)

Where *W*_*ac*_ is the AC of the word feature blob and *Z*_*ac*_ is the AC of the cluster weight. ACs tend to shift intensities of sentiment orientations (polarizations) by regulating augmentations (amplifiers—*W*^*amp*^) and attenuators (de-amplifiers—*W*^*deamp*^). Thus, the final polarized context cluster equation can be defined as:

$$C^{\prime }=\sum_{\;}^{\;}\left[\left(1+W_{amp}+W_{deamp}\right).W^{P}.{\left(-1\right)}^{2+W_{neg}}\right]$$ (6)

Eqs (1)–(6) are only valid for sentences of sufficient lengths and proper structure. Generally, similar mathematical representations used in SentimentR for short texts like tweets and emojis do not work, because the SentimentR feature extraction model requires a minimum n-gram threshold (n-Thres) to accurately label sentiment polarity and intensity.

As EVA features are novel and the subject of this paper, they are described and discussed in greater detail under the **Results** section. In Table 2 below, we show the various features used for comparisons.

**Table 2:** A tabulation of features used in this study.

Feature type	Number of features	Measure
SentimentR	520 (average sentence level) features per journal	Static Sentiment Score
NLP	56 features per journal	Lexical Semantics
EVA	42 (Filtered & Unfiltered)	Relative Polarity Change

In Table 2, as mentioned earlier, the original 21 EVA features are applied on both filtered and unfiltered versions of text, producing a total of 42 features. EVA is compared against SentimentR, which outputs the sentence-wise SA scores (this is the raw sentiment score at the sentence level. EVA’s engineered features captures patterns on these. Hence comparing EVA against SentimentR is meant to demonstrate the value of feature engineering meant to capture patterns of sentiment variation). NLP features are generic and widely/readily available. Hence, EVA also needs to demonstrate utility and superiority in performance benchmarks (in this case, grade prediction based on student journals).

### Multi-Layer Perceptron (MLP) Machine Learning Model

**Fig 7:**
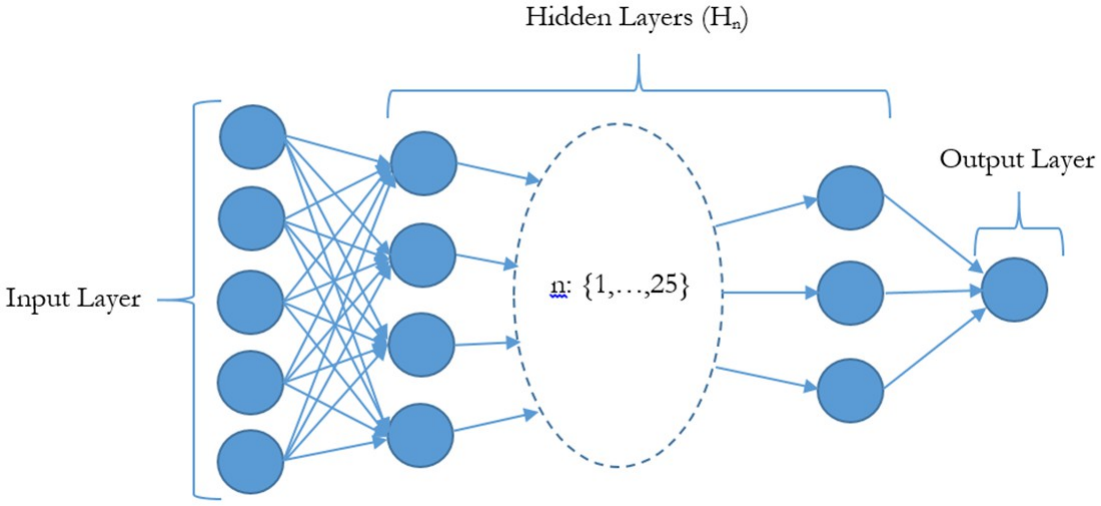
The architecture of the MLP ML model which shows how neurons are interconnected in a “brain-like” synaptic network between input features and output predictions.

We used a MLP model to predict grades from student journals. MLP has an extensible architecture (see Fig 7 for a schema of stacking individual layers from input to output used in this study). Given MLP’s architecture, the output of a single layer becomes the input to the proceeding layer. Such a design enables key features of MLP architectures (e.g. discriminative feed-forward perceptron and loop-back RNNs) to be preserved.

We designed our MLP model to learn through a ReLU neuron activation function framework. Weights of the neurons were corrected from the errors induced by predictions at the output. This model uses the first order stochastic gradient descent (SGD) backpropagation mechanism to iteratively correct for output prediction errors by minimizing error gradients down to specified error tolerance levels (Detailed descriptions and mathematical expressions are given in S1 File).

Although many other AI/ML models exist, the MLP model is a powerful and versatile method of approach used across various scientific studies. Furthermore, the focus here is to determine the information value of the novel EVA features. Hence, we standardized analysis on one primary model.

We use the Design Of Experiment (DOE) criteria [34], choices of calibration methods [35] and errors in observational data to determine our MLP system model parameters. DOE criteria enable us to create a set of systematic procedures for hypothesis testing and choices of calibration methods enable us to test our model against known values called “calibrators”. We used Sensitivity Of Analysis (SOA) methods [36] to determine the best performing parameters in our study based on a predefined behaviour threshold for our model. SOA methods determines how different values of independent variables affect dependent variables under given sets of assumptions. From our approach, we have derived the following parameters for our MLP model:

1. Number of hidden layers = 25
2. Number of hidden neurons per layer: $n_{h=}\frac{2}{3}\left(n_{x}\right)+(n_{y})$ Where n is the number of neurons, h is the hidden layer, x is the input layer and y is the output layer.
3. Activation function = ReLu
4. Solver = SGD
5. Alpha (L2 Penalty) = 1e-5 = 0.00001
6. Maximum number of iterations = 200
7. Error Tolerance = 0.001

Fig 1 shows our implementation model architecture. At the input, individual feature scores from SentimentR, NLP and EVA are fed into the baseline MLP. The MLP was trained at three different depths (i.e. 10, 25 and 45 hidden layers) to give predictions of grades at the output. The predicted grades are then calculated for their F1 accuracy scores (see **Performance Measurements**) from their labelled classifications as either true positive (TP), true negative (TN), false positive (FP) and false negative (FN).

To address model overfitting and slow convergence rates, feature selection was performed before inputting into a linear regression model to obtain a baseline MAE. Each feature was then dropped individually to obtain a set of MAEs for comparison. Features which led to greater MAEs when dropped were deemed to be of higher importance as their exclusion resulted in more errors. This new set of n features was then used as input in the linear model. The input size was varied from 1 to n and their MAEs were compared with the baseline. A smaller amount of top k features with a lower MAE were selected. Changes in MAEs from varying values of k for all feature sets are shown in S2 Fig in S1 File.

To maintain the MLP model’s generalizability when used on different training/testing sets, we used dropouts as a method for deactivating neurons within the neural network that would otherwise overfit the model’s prediction. We applied a dropout rate of 0.20 in alternating succession, from the input to the output layers. This means that if the input layer has 56 activation neurons, a dropout rate of 0.20 at the next alternate successive layer will only activate 45 neurons. The output of the MLP is regressed to a single numerical score–ranking the students learning performance from the experiential course. Additionally, we used the stochastic gradient descent (SGD) with momentum technique as a method to minimize prediction accuracy losses of our MLP model.

### Performance measurements

The F1 score was used to measure accuracy of prediction results obtained from the experiments. F1 scores are popular benchmarks for measuring both contributions of precision and recall as important metrics. Mathematically, the F1 score is given as:

$$F1=\frac{2\times (Precision\times Recall)}{\left(Precision+Recall\right)}$$ (7)

Where

$$Precision=\frac{\left(True\;Positive\right)}{\left(True\;Positive+False\;Positive\right)}$$ (8)

And

$$Recall=\frac{\left(True\;Positive\right)}{\left(True\;Positive+False\;Negative\right)}$$ (9)

**Fig 8:**
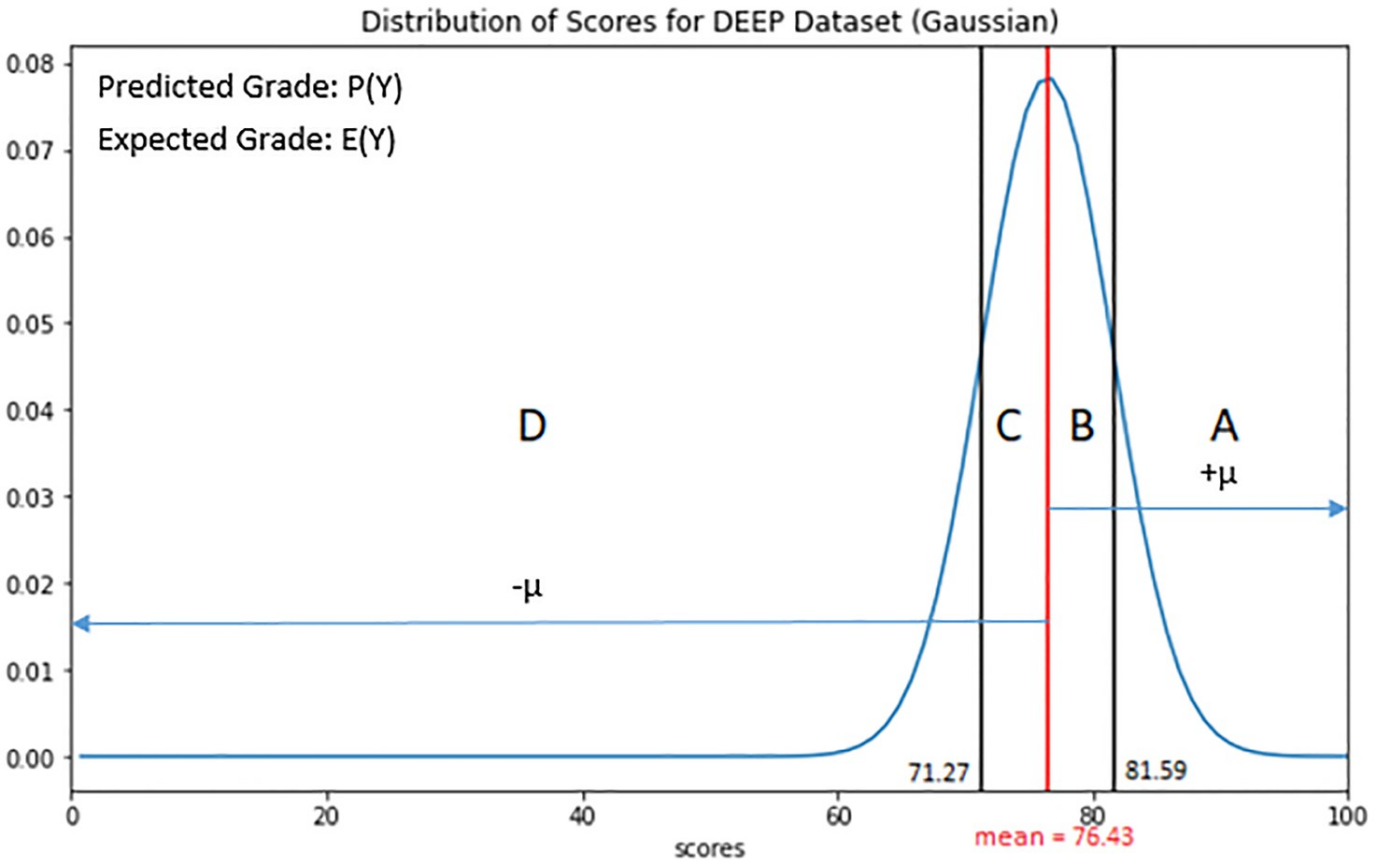
DEEP journal dataset essay grade score distribution.

We determine True Positive (TP), True Negative (TN), False Positive (FP) and False Negative (FN) from DEEP journal essay grade score distributions which is modelled in Fig 8.

Therefore, we define the True Positive (TP), True Negative (TN), False Positive (FP) and False Negative (FN) as:

TruePositive(TP)=PYμ-E(Yμ)≤σPY-μ-E(Y-μ)≤σ (10)

TrueNegative(TN)=σ<PYμ-E(Y-μ)<σσ<PY-μ-E(Yμ)<σ (11)

FalsePositive(FP)=PYμ-E(Yμ)>σPY-μ-E(Y-μ)>σ (12)

FalseNegative(FN)=PYμ-E(Y-μ)≥σPY-μ-E(Yμ)≥σ (13)

Thus, in labelling our prediction results, we approximate *σ~* ± 5 and *μ*~76.

### Cross validation

Training data was partitioned into subsamples and evaluated on K-fold cross validation. Individual subsamples from the K-fold partitions were chosen for validation while the remaining subsamples were retained for training. We set the number of fold partitions as K = 40, and validation was performed over the data source learnt and predicted by the MLP across the Mean Absolute Percentage Error (MAPE) measurement of each run. Mathematically, MAPE is expressed as:

$$\delta_{MAPE}=\frac{1}{N}\sum_{i=1}^{N}\left|\frac{E_{i}\left(x\right)-Y_{i}(t)}{E_{i}(x)}\right|$$ (14)

Where *E*_*i*_(*x*) is the expectation at the output of data input set *i* and *Y*_*i*_(*t*) is the corresponding prediction over *N* total subsamples. The tabulation of the K-fold cross validation used in our experimentation is given in Table 3.

**Table 3:** A table of student grade prediction accuracy scores for the DEEP journals. Predictions made through MLP using the EVA features are the clear winners.

Feature	F1 Score
SentimentR	0.58
NLP	0.76
EVA	0.88

### Institutional review board approval

This specific study was reviewed and approved (Reference Number IRB-2019-10-038) by the Nanyang Technological University institutional review board before the study began.

## Results and discussions

### Emotional Variance Analysis (EVA) features

Sentiment features may capture emotional information correlated to learner motivational states. These sentiment features are generally extracted from reflective journal essay text through detected occurrences of either polarized word *W*_*sgn*_, valance shifters $\frac{{d\nu }_{\epsilon }}{{d\lambda }_{\epsilon }}=C_{W}$, or both [37], [25]. Where the polarization *sgn* of the words *W* in the document (reflective journal essay) *D*, represent either a positive or negative valance *ν*_*ϵ*_; and the shift in valance, which forms a polarized word cluster *C*_*W*_ is given by the relative change *dν*_*ϵ*_ in absolute word valance |*ν*_*ϵ*_| with respect to the change in contextual drift *dλ*_*ϵ*_ per document sentence *σ*_*D*_ = {*W*_1_, *W*_2_, …, *W*_*n*_}. In our method, we identify four types of valence shifters: Amplifiers *α*|*ν*_*ϵ*_|, De-Amplifiers $\frac{1}{\alpha }|\nu_{\epsilon }|$, Negators ¬|*ν*_*ϵ*_|, Adversative Conjunctions *Λ*_*CD*_, where *α* is the amplification factor, ¬ is the negator, *Λ* is the conjunction and *CD* is the contrastive divergence [19].

From these valance shifters, we engineered 21 novel EVA features to address semantic feature resolution losses due to gross over-averaging effects from SentimentR’s [33] aggregate sentiment scores across the entirety of the input [37]. In other words, a lengthy journal written over a long period of time, encompassing multiple paragraphs might have their sentiment scores excessively reduced when summarizing over large datasets.

EVA features were designed through feature engineering to capture patterns of sentiment variability, observed across each student journal. The core principles and assumptions driving EVA’s design are:

1. Gross numerical averaging hides valuable information on variability and consistency. And so, producing a single average sentiment on an entire text or by paragraph clusters, may not be meaningful.
2. Capturing patterns of emotional stability and instability is informative and provide an information-rich feature vector that serves as meaningful inputs for data modelling.

We maintained two versions of journal text. One with all neutral polarity words retained (unfiltered) and the other with all neutral polarity words removed (filtered). The rationale for this step is that although neutral words have a null sentiment score and neutral sentences commonly appear in long text, these “neutral expressions” may provide additional information e.g. in the spacing and intervals of polar word usage [31]. It is not known which version (filtered or unfiltered) is more informative, or if neutral sentences could in general be discarded during SA. Hence, we maintain both filtered and unfiltered EVA features in the feature vector, for a total of 42 features. The filtered features are prefixed with”FS” while the unfiltered ones are prefixed with”UFS" in our feature set. We describe the 21 EVA features as follows:

### 1. Average Sentiment (AVG)

AVG represents the mean sentiment score of all sentences in text. This is the default output from SentimentR at the group structures level. AVG does not capture any patterns but provides a generic average.

### 2 & 3. Longest Happy and Sad Islands (HI, SI)

HIs/SIs are designed to capture persistent and extreme emotive states without signal dilution from other moderate sentiment clusters.

Sentiment scores are dependent on complex interactions between word polarity and modulators per cluster. The sentiment scores between adjacent clusters may be unstable while an average score over clusters will fail to detect this instability. Information on consistent/continuous periods of positive and negative affective states will also be lost. HIs and SIs seek to identify a continuous period of extreme polarity (positive and negative respectively) sentences in text. A happy island (HI) denotes a continuous period of adjacent sentences where sentiment falls within the top 25% ranks of all positive sentiment scores calculated in text. Conversely, a sad island denotes a continuous period where sentiment falls within the bottom 25% ranks of all negative sentiment. Therefore, text with low HI and SI would suggest non-persistence of extreme emotional states.

### 4. Normalized Flip Frequency (FF)

Global averages cancel out interesting variation in data. In SA, a reported global average does not reveal uncertainty or in this case, the emotive “fickleness” of the writer. We designed the flip frequency to capture situations where the succeeding sentence has a strong contrasting polarity with the current sentence under consideration. For example, if sentence 1 has a positive sentiment score but sentence 2 has a negative sentiment score, this is counted as a “flip”. The occurrences of flips are therefore its frequency. Since the flip frequency is count-based such that longer journals could have higher flip frequencies, we normalized this by the total number of sentences in a text as shown in Eq (1):

$$FF=\frac{\sum \left(S_{n}=sgn\cap S_{n+1}=sgn\right)}{N_{sentences}}$$ (15)

Where S_n_ is the sentiment score of the current sentence, S_n+1_ is the sentiment score of the next sentence and N_sentences_ is the text’s sentence count. The numerator captures the two possibilities of a flip.

### 5. Number of Flips (NFF)

This feature is similar to FF. It is an absolute count as it does not include the final normalisation step based on number of sentences.

### 6. Variance of Sentiment Scores (VAR)

VAR calculates the variance of the sentiment scores of all the sentences in the text. The variance gives us an idea about the fluctuation in sentiment of the text. This is a related measure to FF, where the magnitude of variation is also considered (as opposed to only reporting count frequencies in NFF).

### 7 & 8. Hill and Trough Spacing (HS, TS)

While HIs and SIs capture a continuous period of strong emotive state, it may lack sensitivity in the sense it only reports the size of the largest continuous period. Given that there are likely to be other periods of shorter strong emotive states (which we call islands), we designed a measure to check for the intervals or spacings between such islands. Islands are comprised of both hills (positive) and troughs (negative). We define a hill to be the point with the largest magnitude within a HI and a trough to be that within a SI. The spacing between two hills or two troughs indicates the proximity of extreme emotions over a period of time. HS finds the distance between the two longest HIs in terms of the number of sentences between them and is then normalized by the total number of sentences in the text. TS is calculated in the same manner but done for the SIs instead. The equations to compute HS and TS are as mathematically expressed as.


$$HS=\frac{\left|HI_{1}-HI_{2}\right|}{N_{sentences}}$$ (16)

$$TS=\frac{\left|SI_{1}-SI_{2}\right|}{N_{sentences}}$$ (17)

### 9 & 10. Hill and Trough Minimum Sentence Split (HMSS, TMSS)

Similar to HS and TS, these two features count the number of sentences between the top 2 HIs and SIs. However, the final normalisation step is absent.

### 11 & 12. Maximum Sentiment of HI and Minimum Sentiment of SI (MAXHI, MINSI)

MAXHI finds the highest sentiment value in a HI while MINSI finds the most negative sentiment value in a SI. They reflect the strongest points in the periods of extreme emotive states.

### 13 & 14. Variance of HI and SI (HVAR, SVAR)

This is a more specialized form of VAR (see above) in that HVAR and SVAR tracks the fluctuation in sentiment within positive and negative periods of extreme emotive states.

### 15. Moving Average and Root Mean Square Error (RMSE)

Consistency of signal provides evidence of reliability. A consistent emote should not swing between emotive states repeatedly across sentences. And so, comparison of the smoothed sentiment profile of an individual against the original raw sentiment profile should not produce large deviations.

To generate the smoothing function, we define a moving average function across text with a default window size of 3. We then calculate a Root Mean Square Error (RMSE) comparing the smoothened graph against the original.

### 16, 17 & 18. Positive Peaks, Negative Peaks and Peaks Ratio (PP, NP, PR)

To avoid dilutions due to varying periods of positive and negative affective states, we perform a frequency count for positive peaks (PP) and negative peaks (NP) respectively. We then calculate a ratio PR for PP against NP. The ratio acts as a discretized alternative to the AVG.

To provide greater detail, a positive peak is defined to be a point where its sentiment score is higher than that of the points that come before and after it. Conversely, a negative peak is defined to be a point where its sentiment score is lower than that of the points that come before and after it. PP and NP simply counts the number of positive and negative peaks respectively. PR then calculates the ratio of PP to NP.

### 19, 20 & 21. Sum of PP values, Sum of NP values and Average Peak Ratio (SPP, NPP, APR)

PP and NP are frequency-based counts and do not consider the actual values of the positive and negative states. SPP and NPP do: SPP sums up the sentiment values of all positive peaks while NPP sums up that of all negative peaks. APR refers to the ratio of SPP to NPP.

SentimentR and NLP are both quantifications of emotive states in expressed sentiments. Although different in their formulated representations, both SentimentR and NLP rely on fundamental indicators of emotive valance, intensity and polarity to calculate feature scores as a mechanism to discretise and rank continuous sentiment expressions. While SentimentR is a largely sentence and / or paragraph level aggregation of calculated sentiment scores, NLP is a more advanced feature representation vector which includes stochastically defined elements like word frequency, named entities, part of speech tagging, stemming, lemmatization, etc. Relevant tools implementing both SentimentR and NLP include both stochastic and deep learning ML models like decision trees, random forests, regression, k-means, Bayesian, autoencoder, MLP, CNN, ResNet, RNN, Ensemble, etc.

### AI / ML modelling

Given a feature set *F*_*ϵ*_, we wish to predict student performance and measure the applicability of EVA features with ML based approaches to experiential learning courses through prediction accuracies. We leverage on the popular Multi-Layer Perceptron (MLP) machine learning (ML) model to estimate grade score classifiers across the *F*_*ϵ*_ profile and compare the efficacy of using EVA features against other well-known feature set data (e.g. SentimentR and NLP). A notable parallel to our method involves using MLP on bio-inspired problem modelling where the authors used MLP to classify bark textures from localized ternary patterns as one of the discriminating factors of texture features for efficient plant species diagnosis [38].

The main drawback of using traditional sentiment analysis methods (e.g. SentimentR and NLP) is the over-reliance on over-averaged, static and absolute-valued sentiment scores for prediction and classification tasks. As a result, mainstream sentiment feature extraction techniques like SentimentR and NLP are prone to large errors of misclassification and occurrences of outliers [39]. A recent study in [40] uses similar methods and ML models to predict student’s grade scores from both experiential learning course dataset collected from the NTU’s DEEP programme and the Kaggle Hewlett Foundation dataset publicly hosted online. The Kaggle dataset used in [40] contained a lot more essays (totalling 1778) as opposed to the DEEP dataset essays (totalling 37). However, each individual Kaggle essay had significantly lesser word length than their corresponding DEEP counterparts. The authors in [40] implemented the CART DT predictive model and demonstrated that without a Gaussian mask, using NLP features alone on the Kaggle dataset, achieved an accuracy of 82.2%. While using only TAACO features, they were able to optimize accuracies up to 89.2%. Finally, using both TAACO and NLP features, the accuracy dropped to 78.7%. In contrast, using our MLP model with EVA, we show that EVA features alone could achieve an accuracy of 82.7%, and together with NLP, the combined feature set was able to realize an overall accuracy of 89.1%. S1 Table in the S1 File section details the calculated F1 accuracy scores for EVA and NLP on the Kaggle dataset. Additionally, in [40], the authors only obtained an accuracy of 76.5% on TAACO features alone with CART DT, whereas the EVA features as inputs into our MLP model was able to reach a prediction accuracy of 88% as shown in Table 3.

In comparison, studies like [12, 16, 17] extracted features using feature selection algorithms and feature generation approaches from stored data and interactions in massive open online courses (MOOC). In [12], a feature selection algorithm is used to extract the most valuable features for the machine learning (ML) task. The authors used the bio-inspired binary genetic algorithm as a wrapper feature selection on the benchmark dataset from the UCI Machine Learning Repository which contains a collection of education data (including e-learning log files, student marks, admissions / registration data, course details, etc.). A main drawback of their approach is that their feature engineering technique rely on large repositories of heterogeneous datasets which are not easily available. The authors in [16] propose a generative approach to extract learner behaviour features for predicting student dropouts during their learning journey. Their feature engineering approach leverages on machine learning algorithms to determine appropriate weightings of behaviour at each Markovian time window om both recency and correlation. A key limitation in this approach is that although ML models used in their study were powerful, they are not readily explainable. In [17], the authors further develop new measures of click-stream features generated by learners and their interaction in online learning courses by removing identifiable multi-valued inputs. Although such an approach may lower prediction errors by removing outliers, it is unable to discriminate between learners with similar click-stream sequences achieving contrasting grade outcomes.

Our model effectively tackles the problem of accuracy [13] and applicability [18] from two angles. Firstly, a novel set of EVA features which provide excellent information value for machine learning is proposed. Secondly, the novel EVA features are engineered from relative measures of affective sentiment states. Finally, we test our solution on a baseline MLP ML model and compare our results with features extracted from SentimentR and spaCy NLP.

**Fig 9:**
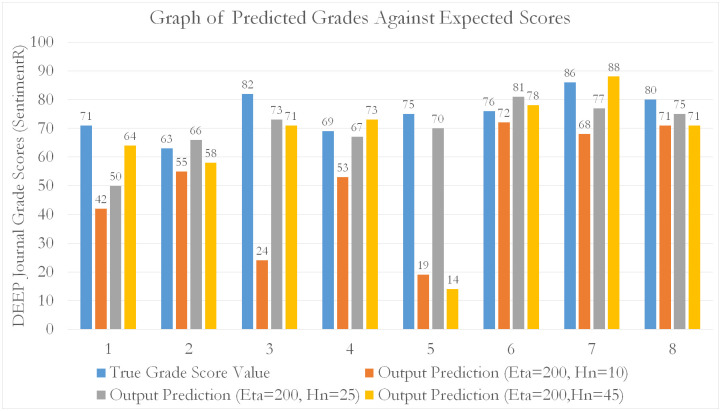
A bar chart showing the distribution of predictions of student grade scores against expected (real) grade scores awarded by course assessors. The predictions were made through MLP, based on the SentimentR features.

**Fig 10:**
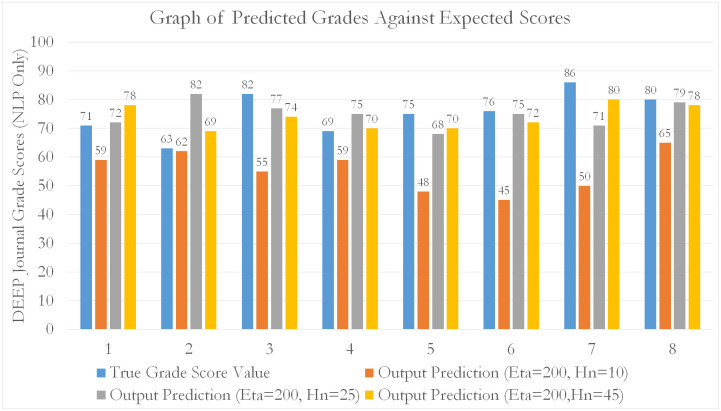
A bar chart showing the distribution of predictions of student grade scores against expected (real) grade scores awarded by course assessors. The predictions were made through MLP, based on the NLP features.

**Fig 11:**
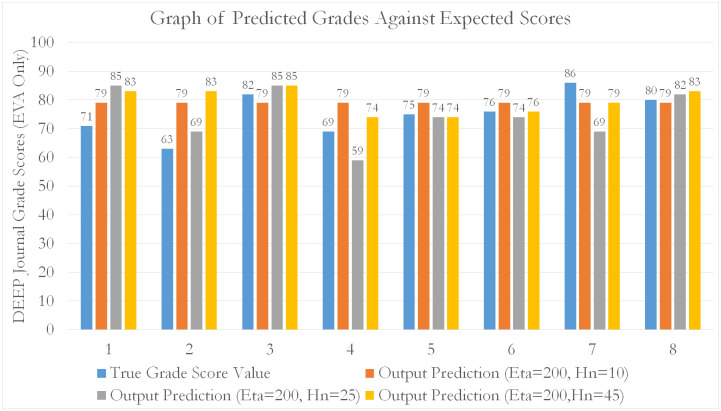
A bar chart showing the distribution of predictions of student grade scores against expected (real) grade scores awarded by course assessors. The predictions were made through MLP, based on the EVA features.

**Fig 12:**
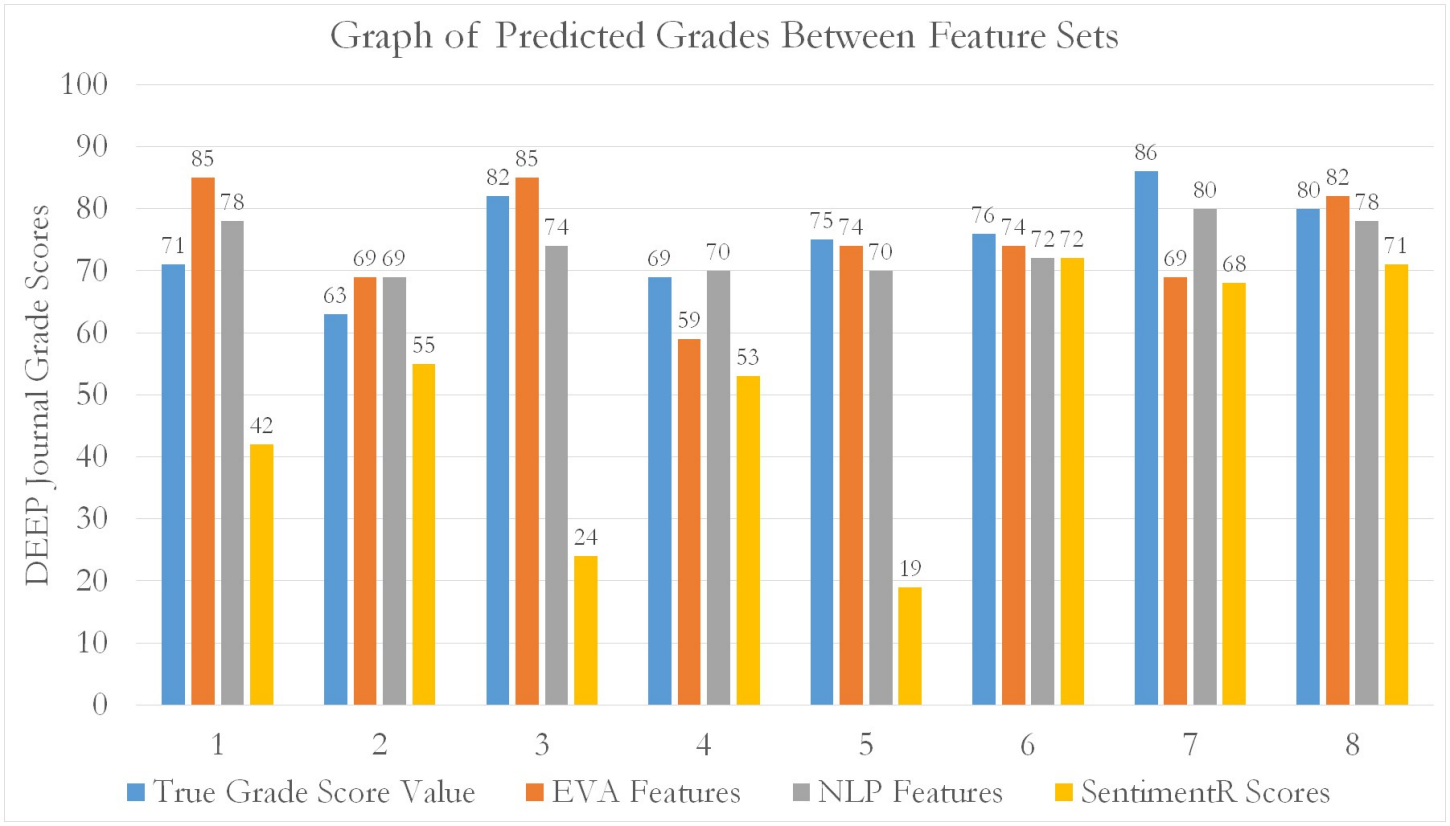
A bar chart showing the distribution of predictions of student grade scores from EVA, NLP and SentimentR feature sets against expected (real) grade scores awarded by course assessors. The predictions were made through MLP.

For all models, an 80–20 train-test split was used to perform k-fold cross-validation. From our tabulated results, it can be seen that EVA features outperform all other feature sets (by approximately 15.8% compared to NLP and 51.7% compared to SentimentR) in predicting student grade scores for the EL course. Generally, as seen from Table 3, the errors decrease as the number of data fold partitions (K) increases. This means that MLP using EVA as predictive features, are able to generalize better across smaller observations (by increasing k). This is evidence by a reduction in predictive variability (diminishing *δ*_*MAPE*_) against increasing folds (smaller subset observations). Additionally, Table 3 shows a more accurate representation of the MLP’s true performance (reduced training bias) when used to predict on EVA features. A tabulation of scores is given in the Tables 3–5. Graphical representations are given in Figs 9–12.

**Table 4:** A table of mean absolute percentage errors (MAPE) over increasing validation folds of the DEEP journal dataset. MAPE drops with successive increments of validation folds.

K	*δ*_*MAPE*_
10	0.514
20	0.452
30	0.367
40	0.289

**Table 5:** A table of ablation test results on the information value contributions to the predictive utility of EVA features on DEEP journals. Unfiltered EVA features have comparably less information value to filtered EVA features.

Method	Precision	Recall	F1 Score
MLP (w/o filtered EVA)	0.667	0.587	0.624
MLP (w/o unfiltered EVA)	0.788	0.891	0.836
MLP (full EVA)	0.844	0.934	0.887

### Parameter influence

The MLP is a complex model that contains several tuneable hyper-parameters. We study the accuracy performance of the MLP by varying the 1) number of hidden layers depth parameter and 2) Error Tolerance. Since EVA features showed the best overall performance, we may keep to using only EVA, while tuning MLP’s hyper-parameters to further improve performance.

**Fig 13:**
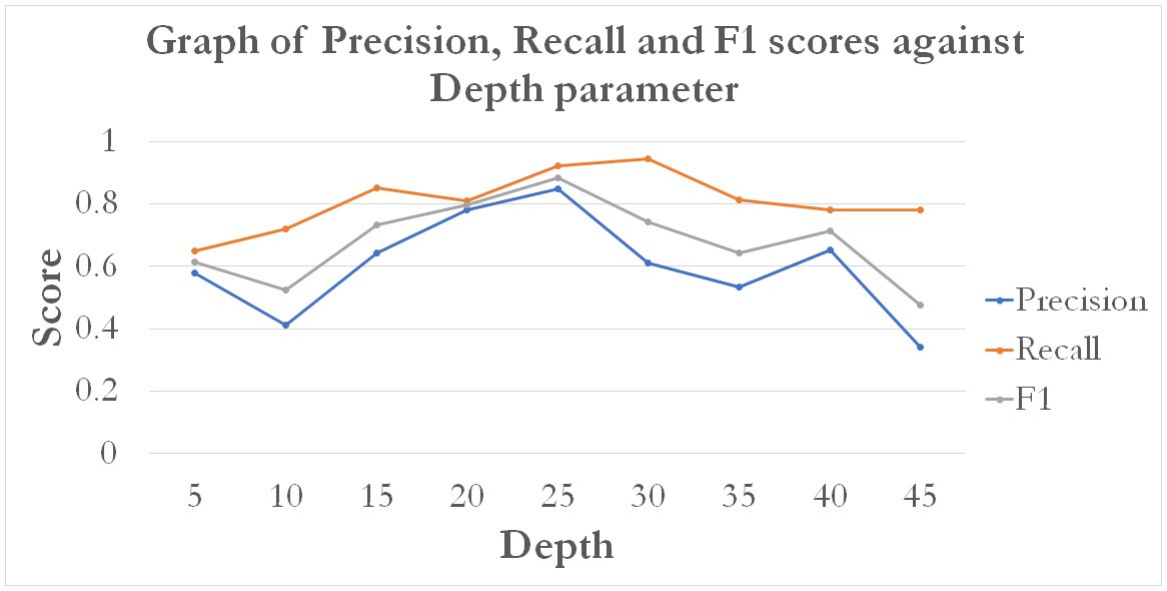
A graph of model performance scores (precision, recall and F1) based on varying MLP depths.

**Fig 14:**
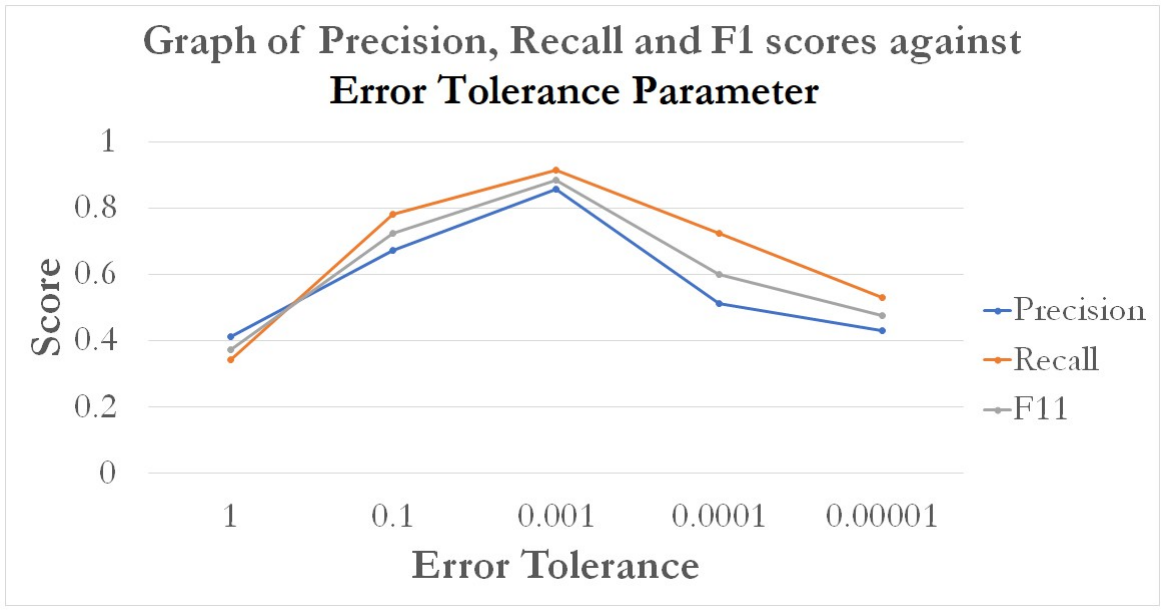
A graph of model performance scores (precision, recall and F1) based on varying MLP prediction error tolerances.

The results show that MLP predictions from EVA features are capable of stabilizing accuracies over a specified range of values. The results of the parameter tuning is given in Figs 13 and 14.

It appears that filtered SA features are more informative. Interestingly, the notion to remove sentences with little emotive content is part of our design, due to the concern that SentimentR’s tendency to average over wide swaths of sentences, including those without emotive content, is likely to dilute signal. This result supports our viewpoint.

## Conclusions

We have developed a set of novel Emotive Variance Analysis (EVA) features that captures patterns and variances associated with emotive content. Firstly, these provide better information value to predicting grades from student journals using ML. Secondly, using a standard MLP model, we find that sentiment analysis features are versatile in predicting student learning performance in an experiential education setting. Finally, we have shown that our new EVA features vastly outperform traditional Natural Language Processing (NLP) features. EVA’s feature engineering process meant to capture patterns of emotional variance is important as it also outperforms the raw sentiment scores from which EVA features are calculated from. Our proposed method using EVA has shown good utility in experiential courses where there is a lot less structure and a lot more autonomy of learning as compared to traditional classroom environments and settings. Although EVA relies on student reflection journals as a form of text analysis to identify and extract sentiments, our model may be automated to analyse translated text from speech. A possible implementation of our model would be in the Massive Open Online Course (MOOC) learning space autonomy, where students may be graded based on skills and knowledge acquired from their interactions and collaborations with online tutoring systems and / or fellow students.

## Supporting information

S1 File (ZIP)

## Abbreviations

AI: Artificial Intelligence
EL: Experiential Learning
EVA: Emotional Variance Analysis
IRR: Inter-Rater-Reliability
ML: Machine Learning
MLP: Multi-Layer Perceptron
NLP: Natural Language Processing
SA: Sentiment Analysis
