# Supplementary material for: Emotional Variance Analysis: A new sentiment analysis feature set for Artificial Intelligence and Machine Learning applications
Source: PLoS One. 2023 Jan 12;18(1):e0274299. doi: 10.1371/journal.pone.0274299 (PMC9836260; doi:10.1371/journal.pone.0274299)
Supplement: S1 File — (ZIP) [file pone.0274299.s001.zip › Supplementary.docx]

**Supplementary**

**Multi-Layer perceptron**

In this architecture, the output of a single layer becomes the input to the proceeding layer. Such a design enables key features of MLP architectures (e.g. discriminative feed-forward perceptron and loop-back RNNs) to be preserved. Mathematically, the MLP can be expressed as:

$$\boldsymbol{Y}\boldsymbol{=}\boldsymbol{AX}\boldsymbol{+}\boldsymbol{B}$$

( 18 )

Where Y is the output, X is the input, A is the node layer transformation matrix and B is the bias. The node layer transformation matrix is a system of weights defined by a layer to layer mapping of confabulations. It is expressed mathematically as:

$$\boldsymbol{A}\boldsymbol{=}\left[ \boldsymbol{H}_{\boldsymbol{1}} \right]\boldsymbol{.}\left[ \boldsymbol{H}_{\boldsymbol{2}} \right]^{\boldsymbol{T}}\boldsymbol{.}\left[ \boldsymbol{H}_{\boldsymbol{3}} \right]\boldsymbol{.}\left[ \boldsymbol{H}_{\boldsymbol{4}} \right]^{\boldsymbol{T}}\boldsymbol{.}\left[ \boldsymbol{H}_{\boldsymbol{5}} \right]\boldsymbol{\ldots}\left[ \boldsymbol{H}_{\boldsymbol{n}} \right]$$

( 19 )

Where H_n_ is the nth hidden layer in an MLP architecture of n layers. The activation function used in our model for individual neurons of the MLP is given by the equation as:

$$\boldsymbol{\sigma}\left( \mathbf{x} \right)\mathbf{=}\mathbf{max}\mathbf{(}\mathbf{0}\mathbf{,}\mathbf{x}\mathbf{)}$$

( 20 )

Which has the characteristic:

$$\boldsymbol{\sigma}\left( \boldsymbol{x} \right)\boldsymbol{:}\left\{ \begin{aligned} \lim_{\boldsymbol{x\to}\boldsymbol{+}\boldsymbol{\infty}} \boldsymbol{=}\boldsymbol{x} \\ \lim_{\boldsymbol{x\to}\boldsymbol{-}\boldsymbol{\infty}} \boldsymbol{=}\boldsymbol{0} \end{aligned} \right.$$

( 21 )

Where sigma(x) is a linearly increasing function as **x→∞** which is continuous and differentiable. A figure of the ReLU rectilinear function is given in Figure S1.

**Figure S1:** A diagram of the ReLU rectilinear function. This activation behaviour enables neurons in the neural network to ignore negative feature values and thresholds, gradually increasing feature values at the input.

The MLP uses an iterative forward and backward pass neuron weight adjustment process to chain known gradients between training layers of the neural network model stack through a method known as the stochastic gradient descent. The backpropagation technique which our MLP model uses is the first order Stochastic Gradient Descent (SGD) with momentum. The objective of SGD is to minimize the cost function C of transforming from a sequence of vectors R^S^. This is done by introducing a perturbation at the upper layer δ-θ to the current upper layer vector ρ such that:

$$\boldsymbol{min\nabla C}{\boldsymbol{(}\boldsymbol{\theta}\boldsymbol{)}}^{\boldsymbol{T}}\boldsymbol{\nabla\theta}$$

( 22 )

For this to occur, a small stepsize α is required to propagate the update rule from the top layers down:

$$\boldsymbol{\theta}^{\boldsymbol{'}}\boldsymbol{\to\theta}\boldsymbol{-}\left( \boldsymbol{\rho\nu}\boldsymbol{+}\boldsymbol{\alpha\nabla C}\boldsymbol{(}\boldsymbol{\theta}\boldsymbol{)} \right)$$

( 23 )

Where the stepsize α is the learning rate, $\rho$ is the memory constant and ν is the velocity vector having represented with the same cardinality as θ. With successive updates, the cost improvement becomes:

$$\boldsymbol{\nabla C}\left( \boldsymbol{\theta} \right)\boldsymbol{=}\frac{\boldsymbol{1}}{\boldsymbol{N}}\sum_{\boldsymbol{i}\boldsymbol{=}\boldsymbol{1}}^{\boldsymbol{N}} \boldsymbol{\nabla}\boldsymbol{C}_{\boldsymbol{x}^{\left[ \boldsymbol{i} \right]}}\boldsymbol{(}\boldsymbol{\theta}^{\boldsymbol{'}}\boldsymbol{)}$$

( 24 )

The first order SGD is both simple and powerful in its approach, and is adopted in our MLP model as an efficient method of convergence to the training output within adequate error tolerance levels.

**Feature Selection**

**Figure S2:** Change in Mean Absolute Error (MAE) scores of the EVA features over varying values of k (the number of top elected features).

**Figure S3:** Diagram of Feature extraction and selection techniques / methodologies used in the Feature Engineering framework.

**Table S1:** A table of accuracy scores for the prediction of student grades using EVA with MLP.

| **Method** | **Precision** | **Recall** | **F1 Score** |
| --- | --- | --- | --- |
| MLP (NLP Only) | 0.803 | 1 | 0.891 |
| MLP (EVA Only) | 0.705 | 1 | 0.827 |
| MLP (NLP + EVA) | 0.803 | 1 | 0.891 |
