# Supplementary figures and images for: Emotional Variance Analysis: A new sentiment analysis feature set for Artificial Intelligence and Machine Learning applications
Source: PLoS One. 2023 Jan 12;18(1):e0274299. doi: 10.1371/journal.pone.0274299 (PMC9836260; doi:10.1371/journal.pone.0274299)

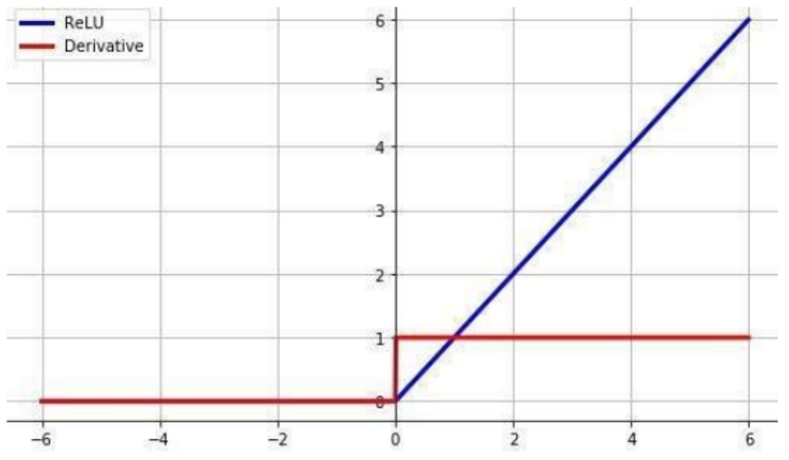

Supplement: S1 File — (ZIP) [file pone.0274299.s001.zip › S1_Fig.tif]

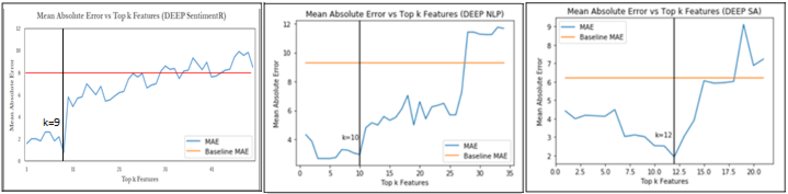

Supplement: S1 File — (ZIP) [file pone.0274299.s001.zip › S2_Fig.tif]

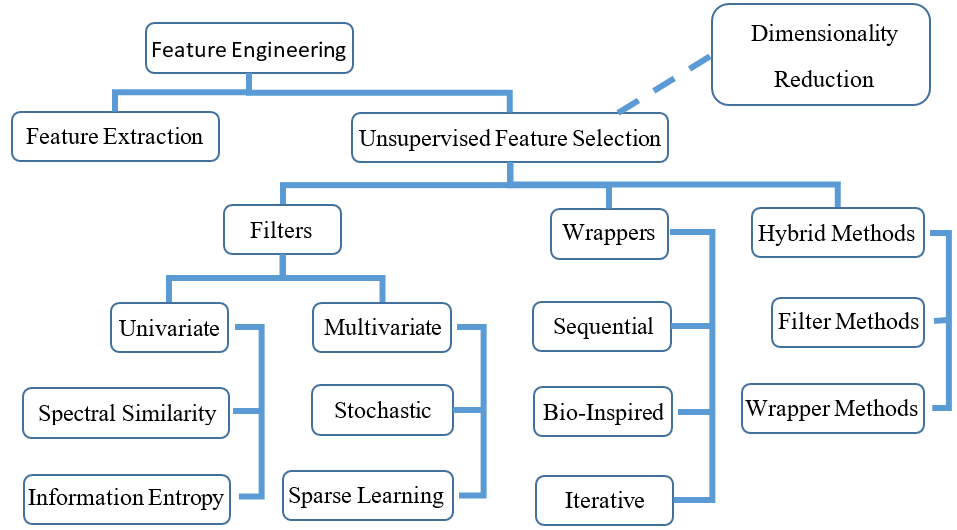

Supplement: S1 File — (ZIP) [file pone.0274299.s001.zip › S3_Fig.tif]
